# Patterns of physical activity in sedentary older individuals with type 2 diabetes

**DOI:** 10.1186/s40842-018-0057-4

**Published:** 2018-04-10

**Authors:** Pearl G. Lee, Jinkyung Ha, Caroline S. Blaum, Kimberlee Gretebeck, Neil B. Alexander

**Affiliations:** 1 Department of Internal Medicine, Division of Geriatric and Palliative Medicine, University of Michigan, Ann Arbor, MI USA; 2 Geriatric Research Education and Clinical Centers (GRECC), VA Ann Arbor Healthcare System, 2215 Fuller Road 11G, Ann Arbor, MI 48105 USA; 3 Department of Medicine, Division of Geriatrics and Palliative Care, New York University, New York, NY USA; 4 College of Nursing, Marquette University, Milwaukee, WI USA

**Keywords:** Diabetes, Physical activity, Physical function, CHAMPS

## Abstract

**Background:**

The Community Healthy Activities Model Program for Seniors (CHAMPS) survey, summarized into weekly caloric expenditures, is a common physical activity (PA) assessment tool among older adults. Specific types of PA reported in the CHAMPS have not been systematically analyzed. We applied latent class analysis to identify the patterns of PA among sedentary older adults with diabetes reported in the CHAMPS survey.

**Methods:**

Latent class models of PA were identified using the CHAMPS survey data reported by 115 individuals aged ≥60 years with type 2 diabetes whom volunteered for a clinical study of PA. Multinomial logistic regression was used to assess independent predictors of a specific latent class, including age, sex, and performance in physical function tests.

**Results:**

Ninety-three percent of the participants were classified into 3 latent classes. Participants in latent class 1 (60.9%) primarily reported domestic-focused activities. Participants in latent class 2 and 3 (19.5% and 19.6%, respectively) reported domestic-focused activities, in addition to leisure-time physical activities and structured exercise activities. Latent class 1, with more women than men (73% vs.27%), had the lowest caloric expenditure, whereas class 3, with fewer women than men (28% vs. 72%), had the highest caloric expenditure (all *p* < 0.001). Latent class 2 had the fastest Timed-Up- and Go (7.65 ± 1.28 s; *p* = 0.03).

**Conclusions:**

Individual PA response in CHAMPS can be categorized using latent class models into meaningful patterns which can inform PA interventions. Customized PA programs should consider the heterogeneity of the activities among sedentary older adults.

**Trial Registration:**

ClinicalTrials.gov Identifier NCT00344240; retrospectively registered 23 June 2006.

## Background

Over 25% of U.S. adults aged 65 years or older have diabetes mellitus, predominately of type 2 [1], and they are at high risks for physical functioning impairment, premature death, and geriatric syndromes [2]. Regular physical activity (PA) is the cornerstone of diabetes management as physical activities not only improve glycemic control [3, 4] but if performed as a part of a lifestyle modification, is effective in maintaining or improving physical functioning among older adults with diabetes [5]. Yet few older adults with diabetes achieve the PA level recommended by professional societies [6].

The American Diabetes Association [7] and the Department of Human Health Services 2008 [8] recommend weekly PA of at least 150 min of moderate-intensity aerobic training and 2 days of strength training for adults with diabetes. Similar recommendations apply to older adults with diabetes who have few comorbidities and are functionally independent [7]. However, adults with diabetes are less likely to be physically active than those without diabetes, with older age and female gender associated with an even lower likelihood of achieving the recommended PA level [6]. Identification of the patterns of PA among sedentary older adults with diabetes, rather than just reviewing a summary score of PA level, may provide insight into why their PA is deficient and then lead to a more informed customized program to enhance their PA.

One of the most commonly utilized tools to evaluate PA in community-dwelling older adults is the CHAMPS (Community Healthy Activities Model Program for Seniors) [9] survey. Respondents report their participation in a number of activities (physical and social) that are commonly performed by older adults, and the time spent in each activity. CHAMPS scores are summarized into estimated weekly caloric expenditure, including calories expended on activities associated with moderate- or vigorous-intensity PA and total overall caloric expenditure. Few studies evaluated the specific types of activities reported in older adults on the CHAMPS, and few identified common patterns of activities in subgroups of older adults with diabetes. Our aim is to sought patterns of PA in subgroups of sedentary older adults with diabetes using a specific analytic tool, latent class analysis, and then evaluate if specific clinical and functional characteristics are associated with these subgroups.

## Methods

### Study design

The current study is an analysis of the baseline characteristics of participants enrolled in “Exercise and Activity Guidance in Older Adults with Diabetes”, a randomized controlled trial to improve PA in sedentary older adults with type 2 diabetes.

### Participants

Inclusion criteria included aged 60 years or older with a diagnosis of diabetes and the ability to walk across a small room without an assistive device. Exclusion criteria included symptomatic cardiopulmonary disease, myocardial infarction in the past six months, daily musculoskeletal pain that hindered exercise, Folstein Mini Mental State Examination < 24 out of 30, participation in regular exercise two or more hours per week, and frequent episodes of low or uncontrolled blood glucose. Participants also underwent a 2D echocardiogram to exclude systolic dysfunction or significant valvular disease, and a maximal treadmill test to exclude exertion-induced ischemia.

Recruitment involved media advertisement, community outreach and mailings to patients with diabetes treated at the Veterans Affairs Ann Arbor Healthcare System and the University of Michigan, both of which provided Institutional Review Board approval. Participants who were screened eligible also had a physical examination with a nurse practitioner. Informed consents were obtained from all participants.

### Measures

The primary measure was PA reported by the study participants using the CHAMPS survey [9]. The CHAMPS survey is a validated 41-item questionnaire specifically designed to document a broad range of activities which vary in physical intensity. Respondents were to report on activities over the past 4 weeks, including the number of times per week and the number of hours per week involved in each activity. Each of these physical activities is assigned a MET (metabolic equivalence task) value consistent with the intensity of effort usually associated with performing the activity, thus allowing calculation of caloric expenditure per week [10].

Covariates included age, sex, BMI (Body Mass Index), race/ethnicity, education, marital status, and key comorbidities common in older adults with diabetes, including hypertension, coronary artery disease, and osteoarthritis. Participants completed the Diabetes Care Profile to assess their attitudes, beliefs, and difficulties with diabetes self-care [11], the Center for Epidemiologic Studies Depression Scale (CES-D) questionnaire [12] to assess depression, and the 12-Item Short Form Health Survey (SF-12) to assess quality of life [13]. Venous blood samples were obtained and processed by the University of Michigan pathology lab, which is certified by National Glycohemoglobin Standardization Program, to determine hemoglobin A1c.

Physical function test included: Timed Up and Go (TUG; time to rise from a chair, walk 10 ft and return to the chair); Six Minute Walk (6 MW; distance walked in 6 min) [14]; and Comfortable Gait Speed over a 10 m walk [15]. Peak Oxygen Uptake (VO_2_, ml/kg/min) was measured during a treadmill walk using a modified Bruce protocol [16]: Starting with a 3 min warm-up at 0% grade, the speed was adjusted to identify the fastest comfortable walking speed for the individual, with subsequent gentle (generally 2% grade) increases at 2 min intervals. Peak VO_2_ is reported because a true maximum VO_2_ is often not possible due to participant comorbidities, fatigue, and motivation [17].

### Data analysis

Among the 41 items in the CHAMPS questionnaire, 28 related to physical activities were used in the analysis; the remainder, which are not strictly physical activities (e.g., social activities, volunteering, hobbies), were not analyzed. The goal was to utilize latent class models to group the 28 physical activity items into smaller numbers of categories for the results to be clinically meaningful. To build the latent class models, we first excluded 9 outlier questions from the analysis because less than 5 respondents (4.3%) participated in each of these activities (i.e., playing basketball, skating, dancing, singles or doubles tennis, jogging, aerobic machine use, yoga, and aerobics). Due to the co-linearity or overlapping of 8 activities, we combined the response to these activities in the subsequent analyses: strength training (*n* = 19) included those reported heavy (*n* = 9) or light strength training (*n* = 14); swimming (*n* = 6) included moderate (*n* = 4) or gentle (n = 6) swimming; walking uphill/fast (*n* = 34) included walking uphill (*n* = 16) or walking fast (*n* = 23); and golf (n = 9) included golfing while carrying golf bag (*n* = 3) or riding a cart (*n* = 8). As a result, we have 15 activities for latent class analyses.

Using the remaining 15 activities, we performed latent class analyses, and estimated all models between 1 and 5 clusters. We determined that the model with 3 latent classes was the optimal model as the bootstrap *p*-value or the goodness of fit test for this latent class model was 0.22, the misclassification rate was small (5.31%), and it has the smallest Bayesian Information Criterion among all the models [18, 19]. We then compared the participants’ characteristics in each of the 3 latent classes, using ANOVA for continuous variables and Fisher’s exact test for categorical variables. We also performed post-hoc tests (Turkey-Kramer pairwise comparisons and proportion test) to localize the differences found between the 3 latent classes. For characteristics that were found to be significantly different in the 3 latent classes (i.e. *p* < 0.05), multinomial logistic models were built to assess the odds ratio of being in one of the three latent classes based on these characteristics.

All statistical analyses were conducted using Latent Gold 4.0 and SAS 9.3 software programs.

## Results

**Fig. 1:**
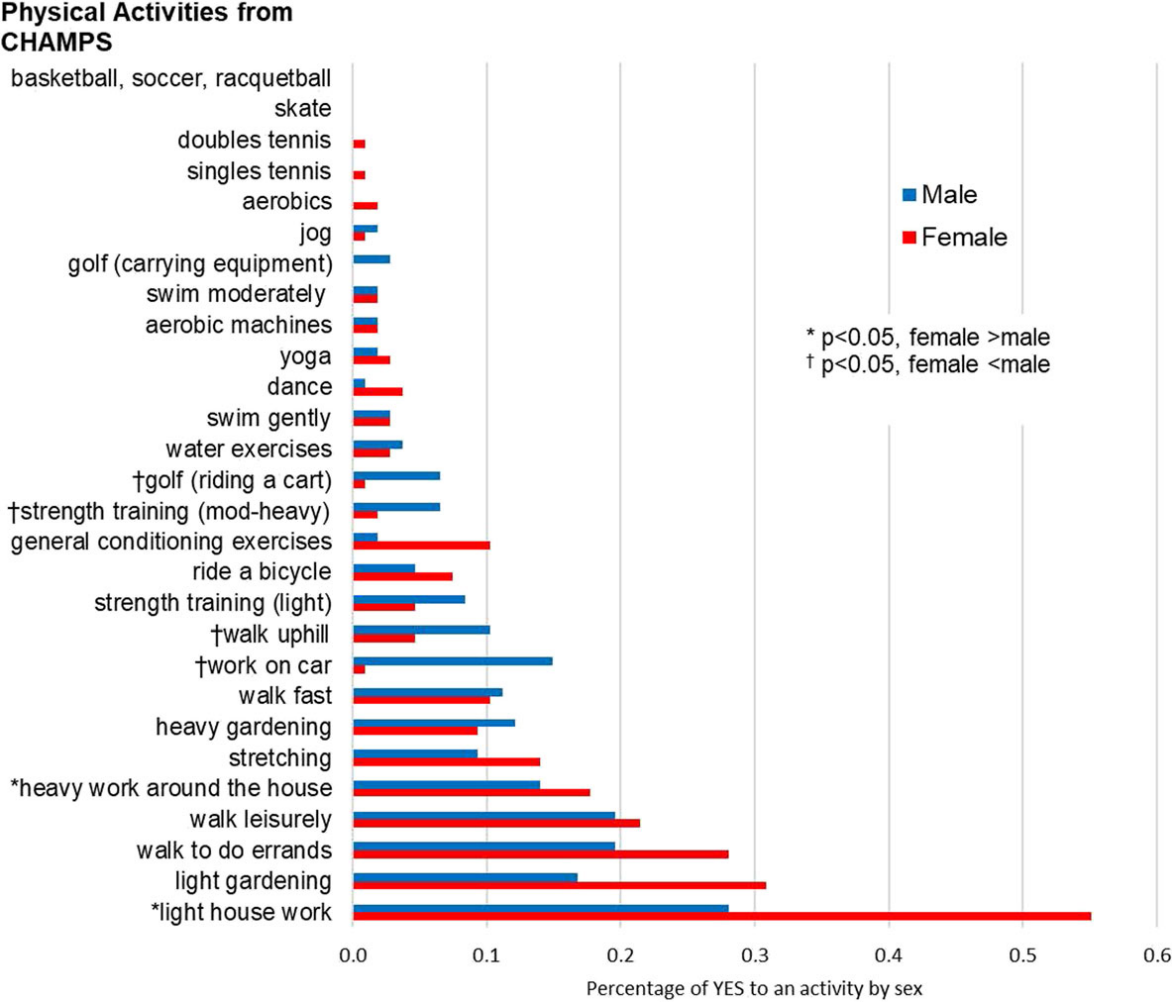
Prevalence of individual physical activity participation reported by study participants, comparing female vs. male. * ^†^ Chi-Square test (Fisher’s Exact test is used if 50% of the cells have expected counts less than 0.5). CHAMPS = Community Healthy Activities Model Program for Seniors survey

Of the total of 115 sedentary diabetes participants, 60% were female and 77% were white, with a mean age of 71 years (See Table 1). The most commonly reported 5 physical activities were light house work, light gardening, walk to do errands, walk leisurely, and heavy work around the house (See Fig. 1). Eighty-three percent of participants reported light house work, making it overwhelmingly the most prevalent activity performed by this population; 51% of participants reported “YES” to the second most prevalent activity, light gardening. Less than 5% of the participants reported participation in dance, yoga, aerobic machines, swimming moderately, golf (carrying equipment), jogging, aerobics, or tennis. No one reported skating, basketball, soccer, or racquetball. Women were more likely than men to perform light or heavy work around the house (*p* < 0.05) (See Fig. 1). Men were more likely than women to work on car, walk uphill, perform strength training, and golf (riding a cart) (*p* < 0.05).

**Table 1:** Characteristics of study participants according to the latent classes of physical activities

Characteristics of participants (*N* = 115)	Mean ± SD or total *N* (%)	Participant Groups by latent classes of physical activity^a^			*P*-values
		Class 1 *N* = 68	Class 2 *N* = 21	Class 3 *N* = 18	
Age, years	70.6 ± 7.1	70.5 ± 7.8	70.1 ± 5.8	71.6 ± 6.7	0.80
Female	66 (60.0%)	50 (73.5%)	10 (47.6%)	5 (27.8%)	< 0.001^α^
White	84 (77.1%)	50 (62%)	17 (81.0%)	15 (83.3%)	0.68
African-American	25 (22.9%)	18 26.5%)	4 (19.1%)	3 (16.7%)	
Some college or more	90 (84.1%)	56 (82.4%)	19 (90%)	15 (83%)	0.74
Married	53 (49.5%)	30 (44.1%)	13 (61.9%)	10 (55.6%)	0.32
BMI (kg/m^2^)	32.7 ± 5.9	33.2 ± 6.5	30.8 ± 4.0	33.9 ± 6.0	0.20
SF-12: Health faire/poor	13 (12.2%)	8 (11.8%)	2 (9.5%)	3 (16.7%)	0.83
Health good/excellent	94 (87.9%)	60 (88.2%)	19 (90.5%)	15 (83.3%)	
Hypertension	94 (83.2%)	58 (85.3%)	15 (71.4%)	16 (88.9%)	0.27
Coronary Artery Disease	14 (12.4%)	7 (10.3%)	3 (14.3%)	3 (16.7%)	0.63
Arthritis	41 (36.3%)	25 (36.8%)	8 (38.1%)	6 (33.3%)	1.00
Lung disease	15 (13.3%)	10 (14.7%)	1 (4.76%)	4 (22.2%)	0.31
Depression (CES-D)	5.84 ± 3.73	5.97 ± 3.72	5.35 ± 3.42	5.89 ± 4.23	0.81
Hemoglobin A1c, %	6.95 ± 1.3	7.03 ± 1.28	6.71 ± 1.08	6.58 ± 0.73	0.28
Using insulin	26 (23.0%)	15 (22.1%)	3 (14.3%)	5 (27.8%)	0.59
Six minute walk (feet)	1313.5 ± 251.9	1311.4 ± 251.8	1345.4 ± 254.5	1334.0 ± 271.1	0.85
Comfortable gait speed (meter/s)	0.95 ± 0.2	0.95 ± 0.2	0.95 ± 0.2	0.95 ± 0.1	0.99
Timed Up and Go test (seconds)	8.70 ± 2.02	9.00 ± 2.11	7.65 ± 1.28	8.74 ± 2.28	0.03^β^
Peak VO_2_ (ml/kg/min)	16.72 ± 4.92	16.59 ± 5.00	17.27 ± 4.64	17.53 ± 5.67	0.73
CHAMPS_TOTAL_ (Kcal/wk)	2482.0 ± 2086.3	1645.1 ± 1505.9	3380.3 ± 1841.7	4363.0 ± 2427.6	<.0001^α^
Frequency per week in any physical activities, times	4.40 ± 2.43	3.15 ± 1.41	6.29 ± 2.03	6.94 ± 2.60	<.0001^α^
CHAMPS_MOD_EXERCISE_ (Kcal/wk)	1250.4 ± 1497.2	593.6 ± 859.1	1989.6 ± 1349.2	2568.5 ± 1759.0	<.0001^α^
Frequency per week of moderate-vigorous physical activities, times	1.72 ± 1.61	0.90 ± 0.95	2.81 ± 1.36	3.61 ± 1.54	<.0001^α^

^a^The 3 latent classes included 107 participants (93% of the total number of participants). Comparison of the 3 latent classes used ANOVA for continuous variables and Fisher’s exact test for categorical variables. Percentages represent column percent. P-values represent comparison of the 3 latent classes

^αβ^ Post-hoc comparison of each latent class used Tukey-Kramer pairwise comparisons (for continuous variables) and proportion test (for categorical variables): α = Both class 1 vs. 2 and class 1 vs. 3 are significantly different (each p < 0.05); β = Only class 1 vs. class 2 is significant (*p* < 0.05)

*SD* Standard deviation, *BMI* Body Mass Index, *CES-D* Center for Epidemiologic Studies Depression Scale questionnaire, *SF-12* 12-Item Short Form Health Survey, *CHAMPS* Community Healthy Activities Model Program for Seniors

**Fig. 2:**
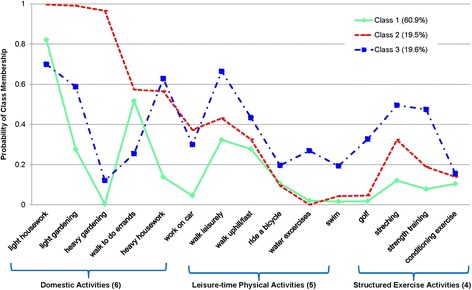
Classification of participants based on prevalence of physical activity participation according to latent class analysis

Ninety-three percent of the participants (*n* = 107) could be classified into 3 latent classes, whereas 8 participants dropped out of the analysis because of missing values in at least one of the PA questions. The 8 participants were similar to the remaining 107 participants with respect to their age, BMI, and performance in 6 MW, gait speed, and TUG (*p* > 0.05). The majority of the participants (60.9%) belonged to latent class 1, which included predominantly *domestic-focused activities* of generally low physical intensity (See Fig. 2). These participants most commonly reported light housework, walking to do errands, light gardening, and walking leisurely, in descending order. Participants in latent class 2 and 3 (*n* = 21 and *n* = 18,19.5% and 19.6%, respectively) had a high probability of reporting domestic-focused activities in addition to *leisure-time physical activities* and structured *exercise activities.*. Class 2 participants were likely to report heavy gardening, walking to do errands, walking leisurely, and stretching. Class 3 participants were likely to report heavy housework, walking leisurely, stretching, and strength training.

Participants in the 3 latent classes were similar in age, race, education, marital status, SF-12, CES-D, Diabetes Care Profile (results not shown), chronic diseases, BMI, A1c, use of insulin, 6 MW, gait speed, and peak VO_2_ (See Table 1). However, there were more women than men in latent class 1 (73% vs.27%) and fewer women than men in class 3 (28% vs. 72%) (both *p* < 0.001). TUG was significantly different among the participants in the 3 latent classes (*p* = 0.03), with the participants in class 2 having the fastest TUG score (7.65 ± 1.28 s), although the participants in all 3 classes had TUG scores consistent with the norms for community-dwelling older adults [20].

Mean total weekly caloric expenditure and moderate-vigorous activity caloric expenditure differed significantly between participants in the 3 latent classes. Latent class 1 had the lowest caloric expenditure and class 3 had the highest (*p* < 0.001). A similar pattern was found in the mean weekly frequency of PA among the 3 latent classes (*p* < 0.01).

Using multinomial logistic analysis, we compared the odds of a participant being classified into one of the 3 latent classes based on age, sex, TUG scores, caloric expenditures and the frequency of PA (Table 2). Compared to class 1, participants in class 2 had lower odds ratio (OR) of having higher TUG score (OR 0.5, 95%CI 0.3–0.9, *p* < 0.02); that is, for every 1 s increased TUG, the odds for being in class 2 verses class 1 decreases 50%. Participants in both class 2 and class 3 were more likely to report more frequent participation in PA (class 2 vs 1 OR 2.8, 95% CI 1.4–5.5, *p* < 0.01; class 3 vs 1 OR 2.6, 95% CI 1.2–5.6, *p* < 0.02). The remaining characteristics were not statistically significant (*p* > 0.05).

**Table 2:** Logistic models comparing the participants in each latent class by their characteristics

Multinomial logistic model	OR of being in class 2 vs. class 1				OR of being in class 3 vs. class 1				Overall
Effect	Odds Ratio	95% Confidence Intervals		*p* value	Odds Ratio	95% Confidence Intervals		p value	p value
Age	1.13	0.98	1.31	0.09	1.16	0.975	1.37	0.095	0.17
Sex (Female vs. male)	0.28	0.04	1.83	0.18	0.14	0.016	1.22	0.075	0.19
Timed get up and go (TUG)	0.49	0.28	0.86	0.013	0.88	0.50	1.53	0.64	0.03
CHAMPS_TOTAL_	1.00	0.99	1.00	0.72	1.00	0.99	1.00	0.41	0.46
Frequency per week, all PA	2.77	1.39	5.51	0.004	2.57	1.18	5.60	0.018	0.01
CHAMPS_MOD_EXERCISE_	1.00	0.99	1.00	0.28	1.00	0.99	1.00	0.67	0.54
Frequency per week, at least moderate intensity PA	1.17	0.38	3.55	0.79	1.99	0.58	6.81	0.27	0.50

*CHAMPS* Community Healthy Activities Model Program for Seniors

## Discussion

In sedentary older adults with diabetes who were recruited for a clinical trial to improve PA, we distilled the large number of (e.g. 28) different physical activities reported in the CHAMPS into a set of 15 items to provide a more meaningful pattern of physical activities. Accordingly, we classified the majority of this cohort (93%) into 3 latent classes based on their CHAMPS responses: class 1- least active, *domestic-focused activities* of generally light intensity; class 2 – more active, mixed *domestic activities, leisure time physical activities and exercises*; and class 3 – most active, with the highest probability of performing *leisure-time PA and exercises* than the other 2 classes. Support of the 3 latent classes is evident in the 3 levels of energy expenditure based on the activities report by each class. Note that summarizing the CHAMPS survey into a single measure, i.e., energy expenditure per week, omits valuable information that can inform future PA interventions. To our knowledge, this unique approach to characterize PA participation using the CHAMPS survey has not been previously reported. Our analyses provided several interesting observations.

Older adults with diabetes who do not meet the recommended PA level (> 150 min moderate intensity PA/week) were heterogeneous in the activities that they participate in. While many were active in a variety of low-intensity, domestic activities, similar to other reports [21, 22], some were participating in moderate-vigorous intense activities. Low-intensity and domestic activities may not have as much fitness benefits as more intensive activities and /or structured exercises, other studies have shown that by just replacing sitting time with any activities, including domestic chores, may improve mortality in adults who are not active [23].

Mortality and the onset of difficulty with activity of daily living may be predicted by poor performance in the Timed Up and Go test [24, 25]. In the present study, the best TUG performance was found in Class 2 participants. Compared to other two classes, participants in Class 2 were more likely to have participated in a variety of different types of activities and of different intensities; they participated in low-intensity and high-intensity domestic activities, leisure-time physical activities, and some structured exercise activities. Our finding suggests that encouraging older adults with diabetes to participate in a variety of different physical activities, rather than selectively participate in a few activities, may improve physical functioning.

In the present study, women were more likely to report domestic/lower intensity PA (Class 1) and were less likely to exercise formally, specifically at a higher intensity (Class 3). This gender difference is consistent with other studies in PA and diabetes [26], and with studies where adult women are less likely to meet guidelines for aerobic PA [27]. Older women, particularly in the present age cohort, may be more likely to fulfill traditional gender roles as homemakers, with more personal and environmental obstacles to structured PA [28]. The implication of our finding is that PA interventions for older adults with diabetes may need to be customized by gender. Note that in larger population studies, older men spent more time in sedentary behavior than women and that even in men aged 71 years or older who exceed current guidelines on PA, more time spent in sedentary behavior is associated with greater mortality risk [29]. Thus, the adverse effects due to prolonged sedentary time may not be completely ameliorated by moderate-vigorous PA. In general, a more thoughtful gender-customized PA plan that limits sedentary time but also includes a range of low to high intensity PA would likely be optimal for older adults with diabetes.

To our knowledge, few studies document detailed information that might be used to tailor PA interventions in sedentary older adults with diabetes. From our data, few of these older adults participate in the types of aerobic and strength training that can lead to benefits in adults with diabetes, such as insulin sensitivity and glucose control [7, 30]. Instead, over 40% of them reported walking leisurely, a low intensity aerobic activity. More research is needed in how to provide aerobic and strength training that is both acceptable to this cohort and is of sufficient intensity to provide health benefits.

The present study supplements the findings by Mooney et al. [31], whom identified five latent classes of physical activity using response from the Physical Activity Scale for the Elderly by adults aged 65–75 years old. They identified that participants in the more active or “athletic” latent classes were more likely to have higher education, higher income and better self-reported health. The present study further applied latent class analysis and identified similar patterns of physical activities in a sedentary older adult diabetes cohort using their response from the CHAMPS, and provided more specific activity information, energy utilization, and physical performance data to describe the three latent classes.

While the select sample is not representative of the general population (i.e., it is predominately white and includes highly educated volunteers for an exercise program), the sample is nonetheless a highly relevant group to be targeted for PA interventions. The self-reported PA level through CHAMPS is not confirmed by objective measurements, but accelerometer-derived estimates omit information on the type of PA individuals participate in and may underestimate energy expenditure through household activities [32].

## Conclusions

In conclusion, use of latent class analysis to classify CHAMPS-acquired PA patterns in sedentary older adults with diabetes can provide insights into future customization of PA interventions.

## Abbreviations

6 MW: Six Minute Walk
BMI: Body Mass Index
CES-D: Center for Epidemiologic Studies Depression Scale questionnaire
CHAMPS: Community Healthy Activities Model Program for Seniors
MET: Metabolic Equivalence Task
PA: Physical activity
SF-12: 12-Item Short Form Health Survey
TUG: Timed Up and Go test
VO2: Peak Oxygen Uptake
